# The Molecular Mechanisms of Cuproptosis and Small-Molecule Drug Design in Diabetes Mellitus

**DOI:** 10.3390/molecules29122852

**Published:** 2024-06-15

**Authors:** Zhaowen Pan, Lan Huang, Yuanyuan Gan, Yan Xia, Wei Yu

**Affiliations:** 1 School of Pharmacy, Xianning Medical College, Hubei University of Science and Technology, Xianning 437100, China; panzhaowen2000@163.com (Z.P.); ganyuanyuan1999@163.com (Y.G.); 2 School of Stomatology and Ophthalmology, Xianning Medical College, Hubei University of Science and Technology, Xianning 437100, China; 15997940576@163.com; 3 School of Biomedical Engineering and Medical Imaging, Xianning Medical College, Hubei University of Science and Technology, Xianning 437100, China; 13797803450@163.com

**Keywords:** cuproptosis, DM, lipoylated proteins, protein–ligand interactions

## Abstract

In the field of human health research, the homeostasis of copper (Cu) is receiving increased attention due to its connection to pathological conditions, including diabetes mellitus (DM). Recent studies have demonstrated that proteins associated with Cu homeostasis, such as ATOX1, FDX1, ATP7A, ATPB, SLC31A1, p53, and UPS, also contribute to DM. Cuproptosis, characterized by Cu homeostasis dysregulation and Cu overload, has been found to cause the oligomerization of lipoylated proteins in mitochondria, loss of iron–sulfur protein, depletion of glutathione, production of reactive oxygen species, and cell death. Further research into how cuproptosis affects DM is essential to uncover its mechanism of action and identify effective interventions. In this article, we review the molecular mechanism of Cu homeostasis and the role of cuproptosis in the pathogenesis of DM. The study of small-molecule drugs that affect these proteins offers the possibility of moving from symptomatic treatment to treating the underlying causes of DM.

## 1. Introduction

Cell death can be categorized into regulated cell death (RCD) and accidental cell death (ACD) [1,2]. RCD includes several forms, including apoptosis, autophagy, pyroptosis, and ferroptosis. In 2022, Tsvetkov et al. published a study in the journal *Science* in which a novel form of cell death caused by copper (Cu) ions was proposed; this was termed cuproptosis [3]. It has been demonstrated that the Cu ionophore elesclomol (ES) increases the intracellular Cu levels and triggers cell death even when it is combined with inhibitors of all known cell death mechanisms, including apoptosis (pan-caspase inhibitor benzyloxycarbonyl-Val-Ala-Asp-fluoromethyl ketone and Boc-Asp (OMe)-fluoromethyl ketone), ferroptosis (ferrostatin-1), necroptosis (necrostatin-1), and oxidative stress (N-acetyl cysteine). This suggests that Cu-induced cell death differs from other known modes of controlled cell death. In addition, tetrathiomolybdate (TTM), a Cu chelator, has also proven effective in rescuing cells from death [3]. A biological system contains two oxidation states of Cu: cuprous (Cu^+^) and cupric (Cu^2+^). Cu^2+^ is a relatively soluble form of Cu that is mostly found in the body, while Cu^+^ is extremely insoluble and more toxic. Cu oxidoreductases facilitate the conversion between the two forms [4]. Cu^+^ can bind to lipoylated protein in the tricarboxylic acid (TCA) cycle, promoting abnormal oligomerization of lipoylated protein. In addition, Cu^+^ can also reduce the level of iron–sulfur (Fe–S) clusters. In both of these processes, a proteotoxic stress response is induced, which ultimately leads to cell death [5,6]. Cu homeostasis is regulated by intricate pathways governing Cu transport and metabolism, involving proteins such as antioxidant protein 1 (ATOX1) [7], ATPase copper transporting alpha (ATP7A), ATPase copper transporting beta (ATP7B) [8], cytochrome C oxidase assembly homolog 17 (COX17) [9], and solute carrier family 31 member 1 (SLC31A1) [10]. Functional imbalances of these proteins are crucial in cuproptosis in cells. Their mechanism of action has been extensively studied in the context of diabetes mellitus (DM), and researchers have revealed the significant roles played by these proteins in the progression and treatment of DM [11,12]. In this review, we examine the existing literature on the molecular mechanisms of cuproptosis, and cuproptosis key proteins, in DM and associated complications. By doing so, we aim to offer insights for future research on cuproptosis in the context of DM, and contribute to the development of therapeutic strategies for DM and its complications.

## 2. Cu Homeostasis and Cuproptosis Mechanism

### 2.1. Cu Metabolism

**Figure 1:**
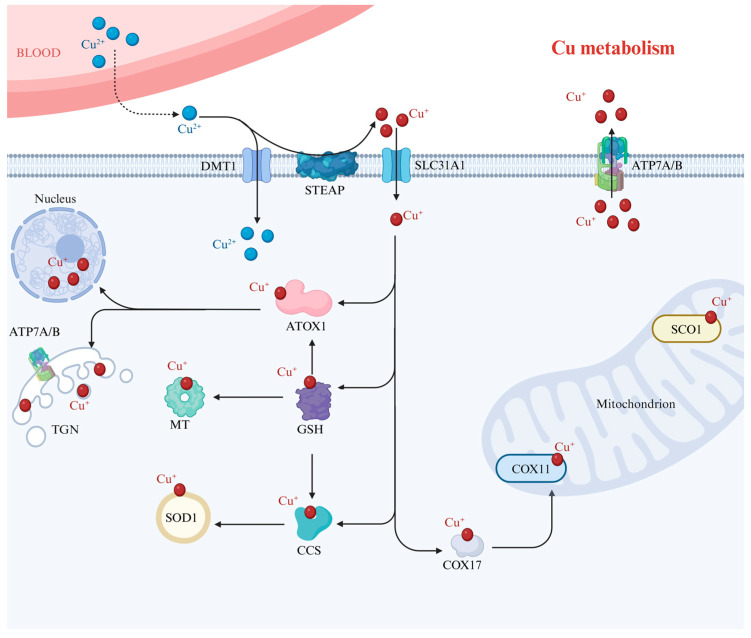
Cu metabolism. Cu^2+^ is reduced to Cu^+^ via STEAP and then crosses the plasma membrane via SLC31A1. Alternatively, Cu^2+^ may cross the plasma membrane directly via DMT1. Cu^+^ is then transported by the chaperone protein ATOX1 into the TGN and the nucleus. GSH can also act as a Cu chaperone protein, binding Cu^+^ and delivering it to MT, ATOX1, CCS, and SOD1. The chaperone protein CCS can also deliver Cu^+^ to SOD1. COX17 delivers Cu^+^ to the mitochondria, which is required for COX11. Together, these processes complete the equilibrium of Cu metabolism. Abbreviations: ATOX1—antioxidant protein 1; ATP7A—ATPase copper transporting alpha; ATP7B—ATPase copper transporting beta; CCS—Cu chaperone for superoxide dismutase; COX17—cytochrome C oxidase assembly homolog 17; COX11—cytochrome c oxidase assembly homolog 11; SCO1—synthesis of cytochrome c oxidase 1; SOD1—superoxide dismutase 1; STEAP—six-transmembrane epithelial antigen of the prostate; SLC31A1—solute carrier family 31 member 1; TGN—trans-Golgi network; MT—metallothionein; DMT1—divalent metal transporter. Created with BioRender.com, accessed on 3 June 2024.

Human biological systems depend on Cu to maintain enzyme activity and transcription factor function as well as to carry out a variety of biological processes, including electron transfer in biomolecules and free radical scavenging [13]. Physiologically, Cu^2+^ is absorbed primarily through the duodenum and small intestine, where most extracellular Cu^2+^ is reduced to Cu^+^ by plasma membrane-localized reductases such as six-transmembrane epithelial antigens of prostate (STEAP), and then transported into the cell by SLC31A1. Additionally, it has been reported that Cu^2+^ can also be imported through the divalent metal transporter (DMT1) [14]. Upon uptake by a cell, it binds to enzymes or Cu-binding proteins, allowing Cu^2+^ or Cu^+^ to be transported to other components of the cell. Additionally, Cu^+^ may be sequestered by metallothionein (MT). First, Cu^+^ in the cytoplasm is bound to ATOX1. It is then transported to the nucleus where it binds to transcription factors and is responsible for regulating gene expression. Additionally, ATOX1 transports Cu^+^ to ATP7A and ATP7B located in the trans-Golgi network (TGN). When the Cu^+^ level is normal, ATP7A and ATP7B are located in the TGN and transport Cu^+^ from the cytoplasm to the cavity within the TGN. But when Cu^+^ levels increase, Cu^+^ is transported intracellularly by ATPase, which fuses with the plasma membrane and releases Cu^+^ [15]. Second, Cu^+^ has been shown to be transported by cellular glutathione (GSH), which regulates Cu^+^ uptake by Cu chaperone for superoxide dismutase (CCS), ATOX1, and MT [16]. Third, in living organisms, Cu^+^ can also contribute to the synthesis of superoxide dismutase 1 (SOD1) under the influence of CCS to maintain the stability of the antioxidant enzyme system [17]. Lastly, COX17 is a soluble protein located in both the mitochondrial intermembrane space and the cytoplasm. Because COX17 is located in both places, it may serve as a Cu^+^ shuttle to the mitochondria for both the synthesis of cytochrome c oxidase 1 (SCO1) and cytochrome c oxidase assembly homolog 11 (COX11) [18]. The Cu transport system plays a key role in maintaining Cu homeostasis and ensuring normal tissue function. The process of Cu metabolism in the human body is shown in Figure 1.

### 2.2. Cuproptosis Mechanism

**Figure 2:**
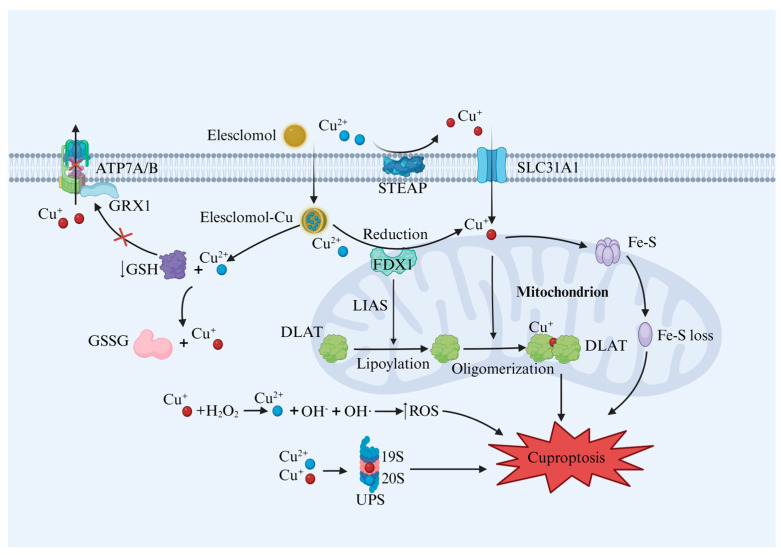
Cuproptosis mechanism. Excessive Cu^+^ entering the cell through SLC31A1 leads to the oligomerization of lipoylated proteins such as DLAT and the loss of Fe–S cluster proteins. The TCA cycle is blocked, leading to proteotoxic stress-induced cell death. In addition, excess Cu^2+^ promotes GSH oxidation, reduces GSH content, increases free Cu^+^ levels and affects ATP7A/B function. At the same time, the increase in intracellular ROS content and inhibition of UPS function result in cuproptosis. Abbreviations: DLAT—dihydrolipoamide S-acetyltransferase; FDX1—ferredoxin 1; Fe–S—iron–sulfur; LIAS—lipoic acid synthetase; STEAP—six-transmembrane epithelial antigens of prostate; GSH—glutathione; GSSG—glutathione disulfide; UPS—ubiquitin–proteasome system; GRX1—Glutaredoxin 1. Created with BioRender.com, accessed on 10 June 2024.

The primary process of cuproptosis relies on the accumulation of Cu within the cell, and is associated with mitochondrial respiration. Researchers have found that Cu homeostasis imbalance, lipoylated protein oligomerization, loss of Fe–S clusters, depletion of GSH, generation of reactive oxygen species (ROS), and inhibition of the ubiquitin–proteasome system (UPS) all contribute to cuproptosis, as shown in Figure 2 [3].

#### 2.2.1. Cu Homeostasis Imbalance and Cuproptosis

Though the body requires sufficient amounts of Cu to sustain growth and development, excessive intracellular Cu^+^ can bind to lipoylated proteins and aggregate, disrupting mitochondrial metabolism. This type of interaction contributes to the promotion of cellular cuproptosis. Cellular cuproptosis can occur in a variety of diseases, including cancer, neurodegenerative diseases, and cardiovascular diseases [19]. Researchers using ES have found that loss of function or reduced function in the ATP7B protein induces Cu accumulation in cells, leading to cuproptosis [3,20]. Understanding this process could pave the way for the development of novel pharmaceuticals or treatment approaches. 

#### 2.2.2. Protein Lipoylation and Cuproptosis

In order to understand the metabolic pathways underlying cuproptosis, the authors of [3] used genome-wide CRISPR-Cas9 loss-of-function technology to screen for loss of function; they identified seven genes associated with Cu-induced cell death including ferredoxin 1 (FDX1), lipoic acid synthetase (LIAS), lipoyltransferase 1 (LIPT1), dihydrolipoamide dehydrogenase (DLD), pyruvate dehydrogenase E1 subunit α1 (PDHA1), pyruvate dehydrogenase E1 subunit β(PDHB), and DLAT. FDX1 is a reductase that reduces Cu^2+^ to Cu^+^, and LIAS is an enzyme involved in the catalytic process of fatty acid synthesis; therefore, FDX1/LIAS controls the lipoylation of proteins upstream. In the TCA cycle, the lipoylation protein dihydrolipoamide S-acetyltransferase (DLAT), a subunit of the pyruvate dehydrogenase complex, is extensively involved in the efficient functioning of the TCA cycle through its pivotal contribution to the conversion of pyruvate to acetyl CoA. Cu^+^ can bind directly to DLAT and promote its oligomerization, thus altering its structure and stability and making it more prone to aggregation, ultimately hindering the activity of TCA [21]. Knockout of FDX1 or LIAS genes intensifies the accumulation of critical TCA cycle intermediates, reducing protein lipoylation and limiting Cu-induced cell death [3]. The Fe–S clusters play important roles in many biological processes, including enzyme catalysis, electron transfer, and the detection of metabolic stress. Cu^+^ has been shown to reduce levels of Fe–S cluster proteins, ultimately leading to protein toxicity and cell death [3]. These findings provide novel insights for us to understand the role of protein lipoylation in cells and the mechanism of cuproptosis. They may also offer innovative targets for developing new drugs or therapeutic strategies.

#### 2.2.3. GSH Depletion and Cuproptosis

Glutaredoxin 1 (GRX1), a 12-kDa protein localized in the cytoplasm and interstitial spaces of mitochondria, exhibits GSH-binding sites and GSH-dependent activity. The interaction of GRX1 with a cysteine-based copper-binding motif in the N-terminal region of ATP7A/7B suggests that glutathionylation of Cu-ATPase may play a regulatory or protective-modification role [22,23]. Glutathionylation and GRX1-mediated deglutathionylation regulate the redox state of the Cu-ATPase cysteine and its binding to Cu^+^. Because GRX 1 requires GSH activity, transport of Cu-regulated ATP7A and ATP7B is impaired when GSH is depleted [24]. Previous research has indicated that GSH may be responsible for facilitating the intracellular movement of Cu^+^ in living organisms. It accomplishes this effect by transferring Cu^+^ to appropriate Cu-chaperone proteins such as ATOX1 and CCS, enabling these proteins to carry out their respective functions [16]. Notably, a reduction in intracellular GSH levels has been shown to correspond to an augmentation in the oligomerization of the lipoylated protein DLAT, rendering tumor cells more susceptible to cuproptosis [25]. N-acetylcysteine (NAC) is a precursor of L-cysteine that results in glutathione biosynthesis. In a recent study, it was found that NAC not only rescued cells from cuproptosis by increasing GSH levels, but also independently diminished intracellular Cu^2+^ uptake triggered by ion carriers [26]. Overall, the role of GSH in cuproptosis is crucial; we may say, therefore, that maintaining intracellular GSH levels and activity is a viable means of preventing cuproptosis.

#### 2.2.4. ROS and Cuproptosis

Cells in the body maintain a dynamic balance between oxidation and antioxidant activity. When this balance is disrupted, oxidative stress can occur, resulting in the production of large amounts of ROS which leads to related cell damage [27]. The Cu^+^ is transformed to Cu^2+^ through a Fenton-like reaction (as shown in Reaction A) in which it reacts with H_2_O_2_ to obtain hydroxyl radical (OH^−^), which causes lipid peroxidation, protein oxidation, and DNA damage, ultimately contributing to oxidative damage to almost all cell components. In addition, Cu^2+^ can react with GSH and transform into highly toxic Cu^+^ and glutathione disulfide (GSSG) (as shown in Reaction B), causing exhausting GSH to clear endogenous ROS and generate Cu^+^ through additional Fenton-like reaction to produce ROS, thus leading to cell death [28]. It has been demonstrated that CuCo_2_O_4_ nanoflowers generated high levels of ROS and reduced the overexpression of GSH in the wound microenvironment, ultimately contributing to bacterial cuproptosis-like death [29]. Meanwhile, ROS increase susceptibility to cuproptosis. For example, studies have found that after encapsulating ES–Cu compounds in ROS-responsive polymers, the compounds are more likely to enter cancer cells, and Cu is readily released, which can trigger cuproptosis and active immune responses [30,31]. In light of these findings, it appears that ROS is involved in cuproptosis, and regulating ROS levels might provide a new avenue to influence cuproptosis occurrence in the future.

Reaction A: Cu^+^ + H_2_O_2_ → Cu^2+^ + OH^−^ + OH

Reaction B: Cu^2+^ + GSH→ Cu^+^ +GSSG

#### 2.2.5. UPS and Cuproptosis

UPS serves as the primary protein quality control system responsible for recognizing and degrading damaged proteins. The UPS consists of several components, including a series of enzymes (E1, E2, E3, and deubiquitinase) and the 26S proteasome (19S regulatory granule and 20S core granule) [32]. There is evidence that Cu^2+^ may be able to bind to the 20S proteasome through non-covalent interactions, thus inhibiting the activity of the proteasome [33]. Additionally, Cu^+^ enhances the ubiquitination of a variety of proteins by affecting the orthosteric activation of the E2-coupled enzyme family UBE2D1–UBE2D4, leading to the accumulation of ubiquitinated proteins [34]. Chen et al. reported that the Hinokitiol Cu complex (HK-Cu) induced striking accumulation of ubiquitinated proteins on A549 and K562 cancer cells, and caused cell death by inhibiting the activity of the 19S proteasomal deubiquitinase (DUB) [35]. Furthermore, the authors of [36] reported that Cu ionophores such as disulfiram/Cu induced ferroptosis and cuproptosis in hepatocellular carcinoma cells, accompanied by a reduction in GSH, an increase in lipid peroxides and a compensatory increase in the solute carrier family 7 member 11, by inhibiting the degradation mediated by the ubiquitin–proteasome. We may say, then, that Cu stabilizes ubiquitinated proteins by interfering with the UPS upstream, thus causing the accumulation of ubiquitinated proteins and implicating the UPS in the cuproptosis process.

## 3. Cuproptosis in DM

**Figure 3:**
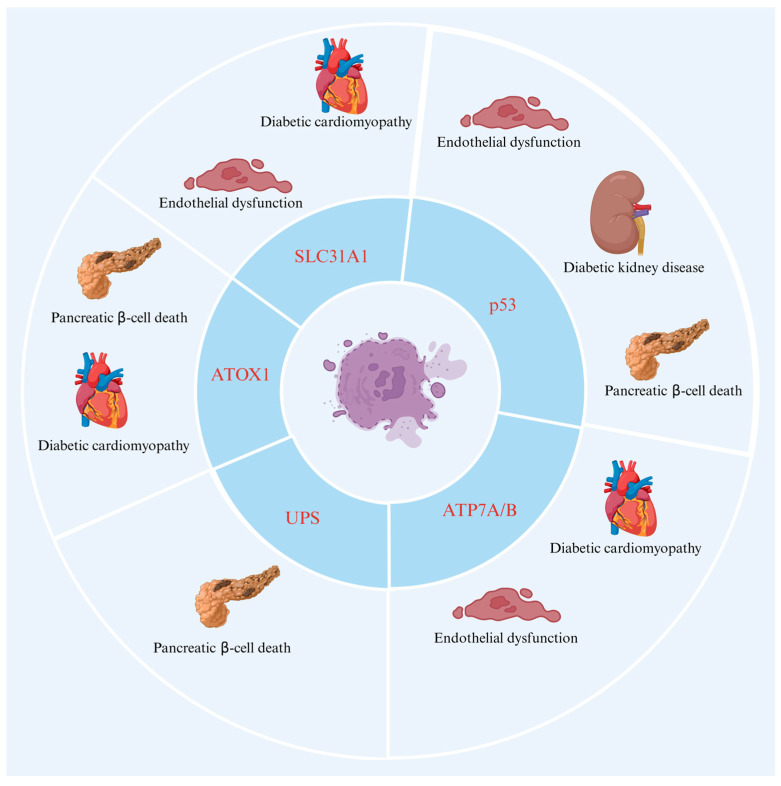
The role of cuproptosis-related proteins in DM and its complications. ATOX1 plays a role in diabetic cardiomyopathy and pancreatic beta-cell death. ATP7A/B is known to be associated with diabetic cardiomyopathy and endothelial dysfunction. SLC31A1 has been implicated in diabetic cardiomyopathy as well as endothelial dysfunction. Pancreatic beta-cell death is also influenced by UPS. Several diabetic complications, including diabetic nephropathy, diabetic cardiomyopathy, pancreatic beta cell death, and endothelial dysfunction have been linked to P53. Abbreviations: ATOX1—antioxidant protein 1; ATP7A—ATPase copper transporting alpha; ATP7B—ATPase copper transporting beta; SLC31A1—solute carrier family 31 member 1; UPS—ubiquitin–proteasome system. Created with BioRender.com, accessed on 3 June 2024.

DM is a complex chronic metabolic disease characterized by elevated blood glucose levels caused by abnormal insulin secretion or function [37]. According to the International Diabetes Federation (IDF), the number of people with DM worldwide reached 529 million in 2021, and this is expected to rise to 1.31 billion by 2050 [38]. Chronic hyperglycemia deteriorates the vascular system, resulting in microvascular diseases such as neuropathy, retinopathy, and nephropathy, as well as macrovascular diseases such as cardiomyopathy [39]. Currently, a comprehensive summary and overview of the signaling pathway of cuproptosis in DM has still not been completed. However, substantial progress has been made in understanding the roles of proteins associated with cuproptosis in DM and its complications; these proteins include ATOX1, FDX1, ATP7A, ATP7B, SLC31A1, p53, and UPS. In addition, researchers have recently found that ATOX1 expression is higher in the liver than in other organs of the body, leading to the conclusion that the liver is the organ primarily responsible for Cu^2+^ metabolism [40,41]. Researchers have also found that there is a high level of expression of the ATP7A protein in the duodenum, suggesting that it may play a role in maintaining the functional homeostasis of this organ under physiological conditions [42]. Additionally, SLC31A1 has been found to be highly expressed in kidneys, testes, eyes, and other tissues, where it is involved in the regulation of Cu homeostasis [43]. In view of the participation and treatment of these regulatory molecules related to cuproptosis in DM, the initiation and regulation mechanism of cuproptosis offers a new perspective for the treatment of DM in the future. It is evident from Figure 3 that the proteins identified above, which are associated with cuproptosis, are also related to DM and its complications.

### 3.1. The Role of Key Proteins of Cuproptosis in DM

#### 3.1.1. ATOX1

ATOX1 is a soluble chaperone protein that is essential for maintaining Cu homeostasis. As Cu^+^ enters the cell through the cell membrane surface protein SLC31A1, ATOX1 transports the cytoplasmic Cu^+^ to ATP7A or ATP7B in the TGN secretion pathway to prevent Cu overload [44,45,46]. Using machine learning to screen cuproptosis gene sets and differentially expressed genes, the authors of [47] identified the cuproptosis-related hub genes of active ulcerative colitis as ATOX1, sulfatase modifying factor 1 (SUMF1), metallothionein 1 G(MT1G), ATP7B, FDX1, and LIAS, thus demonstrating the relationship between ATOX1 and cuproptosis. The effect of Cu^2+^ on the proliferation of pancreatic ductal cells in sheep was also found to promote cell proliferation at appropriate Cu concentrations in another study [48]; however, excess or insufficient Cu^2+^ was found to impair growth, and it was further reported that Cu’s pro-proliferative effect was associated with the activation of the Cu chaperonin 1 ATOX1-dependent MEK/ERK signaling pathway. Another study conducted on streptozotocin (STZ)-induced diabetic mice indicated that Tat-ATOX1 protein reduced blood glucose levels and hemoglobin A1c levels, and also caused changes in body weight. In the same study, it was also shown that Tat-ATOX1 protected pancreatic insulinoma cells such as RINm5F from STZ exposure by inhibiting ROS production, DNA damage, and activating Akt and mitogen-activated protein kinases [49]. Additionally, using immunofluorescence staining, the authors of [11] revealed that the STZ-induced decreased expression of ATOX1 in the left ventricle of diabetic rats was a contributing factor in diabetic cardiomyopathy. These studies indicate that ATOX1 may be involved in the pathogenesis of DM due to its function in regulating Cu^+^ concentration through its binding and transport properties. We may say, therefore, that research on ATOX1 offers new treatment options for DM.

#### 3.1.2. FDX1 

FDX1 is a protein involved in cuproptosis localized to the mitochondrial membrane [50]. Recent studies have identified FDX1 as a regulator of protein lipoylation involved in cellular cuproptosis [51,52]. Additionally, studies have identified FDX1 as a direct target of ES which enhances a distinct form of Cu-dependent cell death [53]. FDX1 first reduces Cu^2+^ to Cu^+^; then, Cu^+^ facilitates the oligomerization of DLAT and expedites the insoluble DLAT and cellular proteotoxic stress. The knockout of FDX1 can indeed reduce the sensitivity of cells to cuproptosis in certain aspects [3]. In addition, FDX1 contributes to Cu^+^ transformation and decreases the Fe–S cluster proteins related to mitochondrial respiration which drive the proteotoxic stress [3]. Fe–S cluster synthesis is dependent on linear mitochondrial FDX1, which stimulates cysteine desulfurase activity and acts as a reducing agent for Fe–S cluster assembly [54]. Proinsulin translation in pancreatic β-cells requires the activity of the Fe–S cluster enzyme CDK5 regulatory subunit associated protein 1 like 1 (CDKAL1). Dysfunctional CDKAL1 can augment misreading of the lysine codon in proinsulin and impair proinsulin processing. Consequently, this process reduces insulin concentration and secretion, and this may facilitate the occurrence and development of DM [55]. Protein FDX1 can participate in the Fe–S cluster assembly process and form a unique cluster, and this small cluster is closely linked to the NOS-containing T2DM network cluster [56]. In a recent study, the expression of cuproptosis genes was analyzed in a healthy population and in diabetic samples. Using the String online database, Cytoscape v3.9.1 (https://cytoscape.org/), and Wilcox analyze, it was found that the cuproptosis gene FDX1 is associated with diabetic immune invasion [57]. Consequently, it is important to study how the structure of FDX1 binds Cu^2+^ and inhibits the formation of Fe–S clusters, in order to target FDX1 and prevent cuproptosis in DM.

#### 3.1.3. ATP7A and ATP7B

As pivotal proteins in Cu^+^ transport, ATP7A and ATP7B collaborate to maintain Cu^+^ concentration balance. Disruptions in Cu homeostasis negatively affect various bodily functions, as exemplified by Menkes’ and Wilson’s diseases, both of which arise from ATP7A/ATP7B gene mutations. Mutations in the ATP7A gene cause functional abnormalities, leading to Cu^+^ accumulation in the pancreas, β-cell apoptosis, and DM progression [20]. Studies have found that rats with DM induced by STZ exhibit lower ATP7B levels than normal rats, with no significant change in ATP7A expression. Due to reduced expression and altered localization of ATP7B in the diabetic myocardium, Cu^+^ efflux from the cells is impaired, which may allow for a local imbalance of Cu [11]. A comparative gene-expression analysis of healthy and diabetic individuals revealed significant downregulation of cuproptosis protein ATP7B, DLAT, glycine cleavage system protein H (GCSH), LIPT1, and DLD in diabetic patients, along with significant increases in the dihydrolipoamide branched chain transacylase E2 (DBT) gene [57]. In mice with type 1 diabetes mellitus (T1DM), a decreased level of ATP7A protein can result in impaired SOD3 activity and vascular oxidative stress and endothelial dysfunction [58]. In addition, a study by Sudhahar V. et al. found that ATP7A expression was significantly downregulated in T2DM patients’ vessels as well as in high-fat diet-induced or db/db T2DM mice’s vessels. Through the activation of Akt2 in VSMCs, insulin prevented the degradation and ubiquitination of ATP7A and its translocation to the plasma membrane, which protects against the endothelial dysfunction associated with type 2 diabetes mellitus (T2DM) [12]. The authors of [59] found that, in T2DM patients and in mice, ATP7A expression was significantly reduced in exosomes, impairing wound healing and angiogenesis, but exercise training restored the angiogenic effects by increasing ATP7A levels. Furthermore, another study reported that, in liver cancer cells, levels of ATP7A and ATP7B mRNA were significantly elevated when stimulated by free fatty acids to establish an insulin resistance model, and that levels of ATP7A were significantly increased when stimulated by free fatty acids for 24 h, while levels of ATP7B were slightly decreased, and Cu content in the cell was reduced [60]. Further research is required to determine whether ATP7A/B regulates intracellular Cu homeostasis or cuproptosis in DM and whether it contributes to this disease.

#### 3.1.4. SLC31A1

SLC31A1 is mainly localized in the plasma membrane. SLC31A1 is widely expressed and has been shown to be a key high-affinity protein for Cu^+^ [61]. Researchers have reported significantly elevated Cu^2+^ levels in both the serum and myocardial tissue of diabetic mice compared to controls. Furthermore, excessive Cu^2+^ in DM upregulated SLC31A1 expression through ATF3/SPI1, thereby mediating Cu^+^ accumulation in cardiomyocytes [62]. In the vascular tissues of diabetic db/db mice and endothelial cells exposed to high glucose, elevated levels of absent, small or homeotic 2-like protein (ASH2L) have been shown to activate transcription of the reductase STEAP, which enhances the transport of Cu^+^ into the endothelial cells by SLC31A1, thus causing oxidative stress and inflammation and leading to endothelial dysfunction [63]. It is evident that the regulation of SLC31A1, which controls Cu^+^ in diabetic patients, offers novel insights and therapeutic strategies for DM treatment.

### 3.2. The Role of Potential Proteins of Cuproptosis in DM

#### 3.2.1. p53

The p53 gene is a human oncogene involved in the regulation of apoptosis, aging, DNA repair and glucose metabolism. In recent years, p53 has been shown to have an essential effect on cuproptosis regulation [64]. Researchers have recorded significantly increased ATOX1 levels in p53-inactivated cells, suggesting that p53 is a negative regulator of ATOX1, and that regulation of p53 expression is crucial for intracellular Cu^+^ transport [65,66]. In addition to blunting glycolysis and promoting metabolism in the TCA cycle [67], p53 may also have notable effects on other pathways involved in cuproptosis, including increasing the instability of Fe–S clusters and reducing the production of GSH. The authors of [68] reported that, in diabetic mice, excess-protein O-GlcNAcylation increased p53 expression and led to coronary microvascular disease, while inhibition of p53 restores capillary density and endothelial cell apoptosis, and decreases cardiac damage, after ischaemia/reperfusion. In another study, STZ-treated diabetic mice and diabetic patients showed marked upregulation and activation of p53, and blocking p53 led to attenuated miR-214 induction in diabetic kidney disease [69]. Using a T1DM mouse model, the authors of [70] found that the p53 inhibitor pifithrin-α prevented the progression of DM-induced cardiac remodeling and dysfunction. The early growth response 1 gene (Egr1) is stimulated by hyperglycemia, and Egr1 promotes p53 transcription which is necessary for apoptosis, migration, and angiogenesis in retinal endothelial cells [71]. Because p53 regulates Cu homeostasis and TCA cycling, as well as its influence on DM and its complications, therefore, we hypothesize that p53 might be associated with cuproptosis in DM.

#### 3.2.2. UPS

The UPS is responsible for cellular value addition, differentiation, signal transduction and selective protein hydrolysis processing. UbcD1 is one of 40 E2 ubiquitin-conjugated enzymes that interact with many E3s [72,73,74] and is a Cu protein whose immediate mammalian homolog, UBE2D2, loses some activity when its Cu-binding region is mutated. This implies that UbcD1 activity may be affected by Cu^+^ levels under normal conditions [75]. The authors of [76] examined the role of UbcD1 and found that overexpression of UbcD1 did not produce any recognizable defects, whereas inhibition of UbcD1 led to a decrease in SLC31A1 expression, suggesting that UbcD1 promotes the stability of SLC31A1 rather than promoting proteasomal degradation [76]. We may say, then, that UPS plays a significant role in Cu homeostasis in vivo. Nevertheless, excessive accumulation of Cu can also enhance proteasome damage. In this regard, it is noteworthy that Cu^2+^ can damage the gate system of the 20S proteasome, which is an essential target for protein catalysis. This damage instigates the proteasome suppression and 26S proteasome degradation mediated by ROS [77]. In addition, some experimental studies have highlighted that UPS is related to insulin production, and restriction of UPS was shown to diminish insulin production by the authors of [78]. Researchers have also shown that small ubiquitin-like modifier (SUMOylation) is a post-translational modification of proteins. Its associated ubiquitin–proteasome system has been shown to mediate protein quality control in the heart, and to play an important role in the proteotoxic environment of the heart. Specifically, ubiquitin-conjugating enzyme E2 (Ubc9) is the only SUMO-E2 enzyme whose expression positively regulates autophagy in cardiomyocytes with potential cardioprotective effects [79]. Accumulation of ubiquitinated proteins has also been recorded in cultured pancreatic islets and β-cells, and inhibition of proteasome activity has been observed in the pancreas of Zucker diabetic rats, but whether this was the cause or consequence of β-cell damage remains unclear [80]. The authors of [81] used microarray analysis to explore the pancreatic islet transcriptome of patients with T2DM, and found that more than 1000 genes were differentially expressed in the pancreatic islet transcriptome of diabetic patients, compared with non-diabetic patients, and that these genes were associated with the UPS. Reduced proteomic activity was also recorded in the pancreatic islets of diabetic patients, and this was associated with defects in pancreatic β-cell function [81]. In addition, proteasome dysfunction has been associated with obesity in humans, and has also been recorded studies on islet misfolded proteins in diabetic rats [82]. In light of the above, we hypothesize that UPS may affect DM through the cuproptosis pathway.

## 4. Cuproptosis Drugs

### Advances in Research into Small-Molecule Drugs Targeting Cuproptosis

Pharmaceutical substances that impact the cuproptosis protein such as methotrexate, olsalazine, mitotane, nicotinamide adenine dinucleotide (NADH), radicicol, dihydrolipoic acid (DHLA), and dexmedetomidine (DEX). Chen Y. et al. conducted molecular docking experiments on methotrexate and olsalazine with the FDX1 protein and found that methotrexate binds with FDX1 through Arg 74, Thr 114, His 116, Tyr 142, Glu 169, and Val 171, resulting in a binding energy of −9.8 kcal/mol, while olsalazine binds with FDX1 through Leu 140, Tyr 142, Val 171, Arg 74, and Thr 114 with a binding energy of −9.1 kcal/mol [83]. Hongfang Li et al. carried out analysis using AutoDock and found that mitotane binds to multiple amino acid sites of FDX1 (including Leu460/209, Phe458, Val35/353, and Ile84) with a binding affinity of −8.1 kcal/mol, while NADH, radicicol, and DHLA bind to the amino acid sites of DLAT (including Phe48/35/32, His168, Met47, Asn39, and Gln31) with binding affinities of −8.1 kcal/mol, −6.4 kcal/mol, and −5.3 kcal/mol, respectively [84]. Researchers have also found that DEX is effective in treating brain ischemia/reperfusion injury by decreasing the levels of SLC31A1, FDX1 and LIAS and the aggregation of DLAT, while increasing the production of ATP7B. Guo Q. et al. used molecular docking analysis to find that DEX can tightly integrate with SLC31A1 and FDX1. Furthermore, when FDX1 is overexpressed, these effects of DEX are attenuated. It may be suggested, then, that FDX1 is associated with the regulatory pathway of DEX [85]. Yang X. et al. used the Enrichr database to analyze small molecules and drugs targeting the key cuproptosis protein in temporal lobe epilepsy (TLE), and found that chlorzoxazone and piperlongumine were the most likely to target cuproptosis genes and thus inhibit cuproptosis [86]. In addition, Jinke Huang. et al. used Enrich to reveal that latamoxef, vitinoin, clomipramine, chlorzoxazone, glibenclamide, pyruvic acid, clindamycin, medrysone, and flavin adenine dinucleotide all might affect genes associated with cuproptosis in ulcerative colitis [87]. The interactions between drugs and proteins are summarized in Table 1. 

Through advances in science and technology, researchers have acquired a range of techniques to uncover the process of cuproptosis and identify potential pharmacological targets. Among these techniques, database screening and molecular docking analysis are particularly significant in current drug research. Presently, most drug studies on cuproptosis are in the fields of cancer and mental diseases. Drug studies on DM are still lacking. Based on ideas used in research into other diseases, we can quickly screen DM therapeutic drugs with an anti-cuproptosis process and design experiments to verify their effects by recommending drugs or consulting databases. In addition to these methods, we can also use high-throughput screening and structural biology to make breakthroughs in the treatment of DM-induced cuproptosis. 

## 5. Conclusions and Perspectives

In this review, we have comprehensively examined the mechanisms underlying cuproptosis, as well as the signaling pathways and proteins associated with it, in DM. As mentioned earlier, cuproptosis is primarily a result of the interaction between Cu and proteins during the TCA cycle. Several key proteins implicated in cuproptosis, such as ATOX1, FDX1, ATP7A/B, SLC31A1, p53, and UPS, exhibit strong associations with DM. ATOX1, which facilitates Cu transport within cells, has potential as a therapeutic target for treating DM. A mutation in ATP7A/B affects the excretion of Cu^+^ from the cell, resulting in an increased rate of pancreatic cell death in DM, whereas mutations in FDX1 may disrupt the synthesis of Fe–S clusters, resulting in metabolic disorders. In cardiomyocytes, excess Cu^2+^ upregulates SLC31A1 expression, disrupts Cu homeostasis and promotes cuproptosis. Among P53’s many regulatory functions, it modulates GSH levels and suppresses ATOX1 expression, and failure of UPS may lead to impaired insulin production. Follow-up studies should utilize computer analysis and other means to search for drugs that inhibit cuproptosis through the regulation of the proteins involved in cuproptosis.

Recent trends indicate that DM is on the rise, resulting in a greater need for effective treatment in society. A growing body of evidence also indicates that cuproptosis contributes to the development of DM. In preclinical trials and animal experiments, inhibition of Cu metabolism has been shown to improve DM and its complications, thus offering a new therapeutic approach for the treatment of DM. It is still necessary to improve cuproptosis in DM, because many known cuproptosis-related targets have not yet been directly verified in DM models. It is important to focus research on cuproptosis and its mechanism of action in the context of DM in the future, and the mechanism underlying cuproptosis in DM should be explored further.

## Figures and Tables

**Table 1:** Drugs targeting cuproptosis-related proteins.

Drug Name	Drug Structure	Targeting Protein	Binding Affinity	Binding Sites	Combined Score	Mechanism of Action	Refs.
Methotrexate	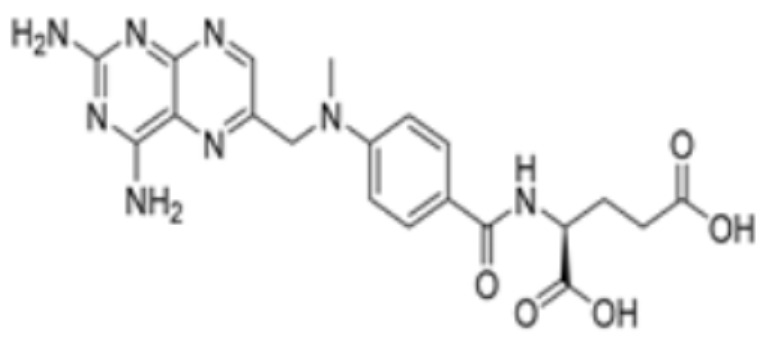	FDX1	−9.8	Arg74, Thr114, His116, Tyr142, Glu169, Val 171	/	Treats IBD, may have some effect in combating cuproptosis, due to its ability to tightly bind to FDX1.	[83]
Olsalazine	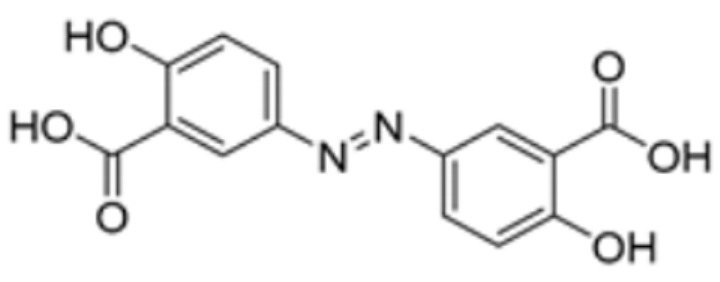	FDX1	−9.1	Leu140, Tyr142, Val171, Arg74, Thr 114	/		[83]
Mitotane	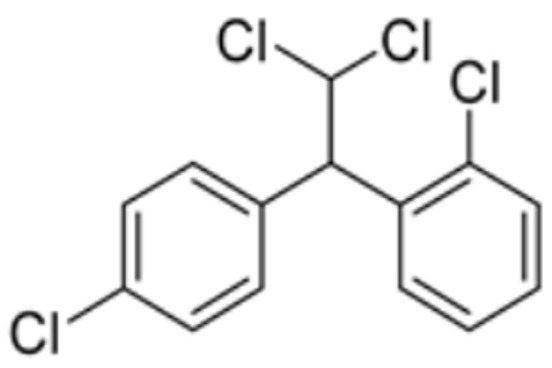	FDX1	−8.1	Leu 140, Tyr 142, Val 171, Arg 74, Thr 114	/	Found to bind to multiple amino acid sites of FDX1.	[84]
Nicotinamide adenine dinucleotide	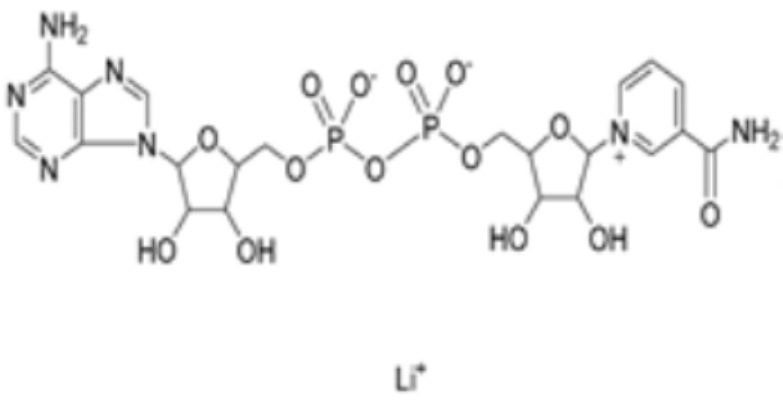	DLAT	−8.1	Phe48/35, His168, Leu27	/	Found to bind to multiple amino acid sites of DLAT.	[84]
Radicicol	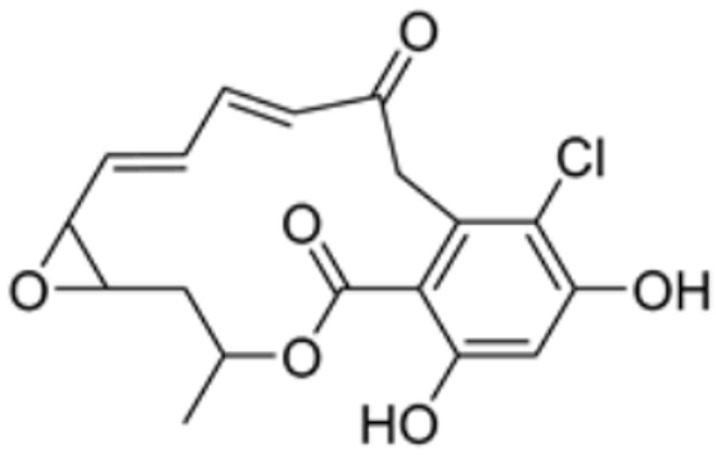	DLAT	−6.4	Phe35/32,His168, Leu164, Gln167, Thr44, Lys173, Met47	/		[84]
Dihydrolipoic acid	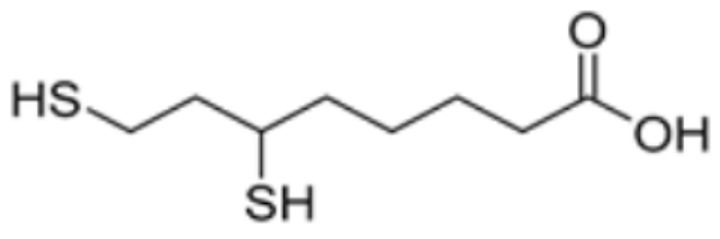	DLAT	−5.3	Phe35/48, Asn39, Gln-31	/		[84]
Dexmedetomidine	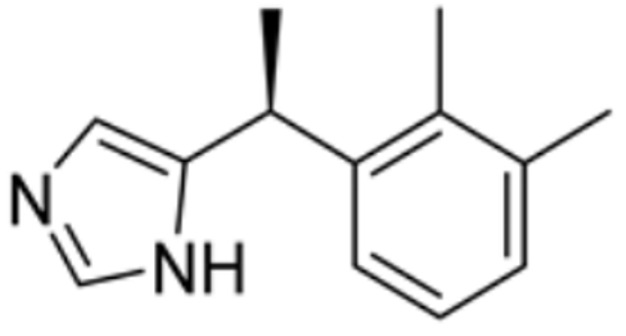	FDX1, SLC31A1	/	/	/	Reduction in FDX1 and SLC31A1 levels prevents cuproptosis.	[85]
Chlorzoxazone	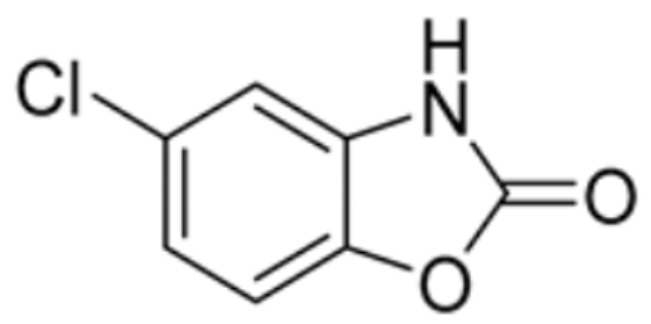	LIPT1, FDX1, DLD, PDHB	/	/	301.8211	Suppression of LIPT1, FDX1 expression and DLD to impede hippocampal neuron cuproptosis in TLE.	[86]
Piperlongumine	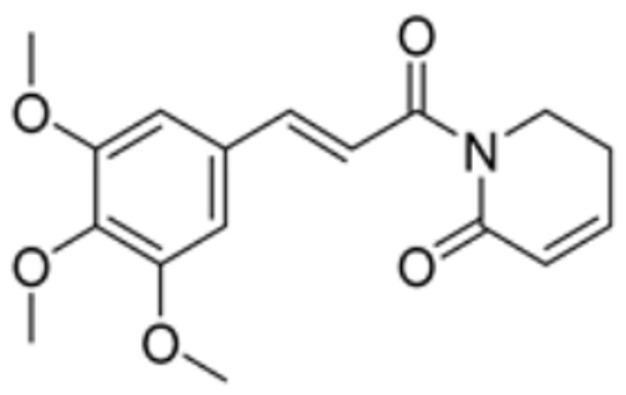	LIPT1, PDHB	/	/	309.921	Suppression of LIPT1 and PDHB expression to impede hippocampal neuron cuproptosis in TLE.	[86]
Llatamoxef	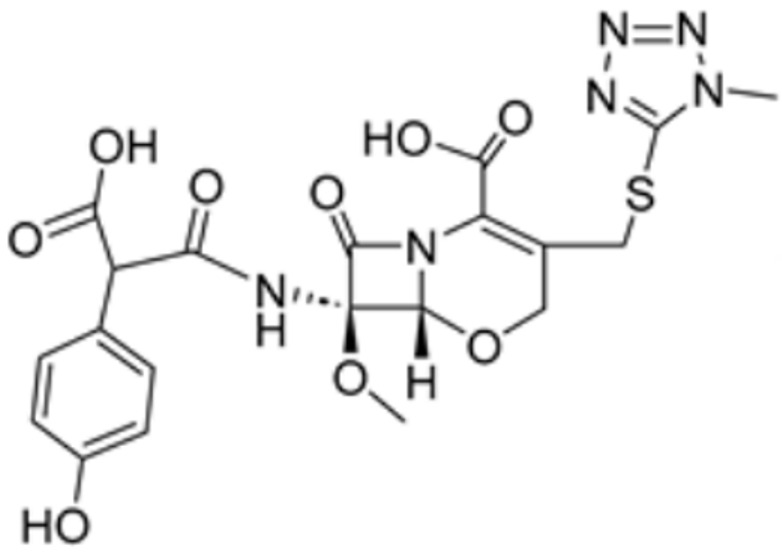	PDHA1, FDX1, DBT, DLAT, LIAS	/	/	173.328	Potential cuproptosis-related gene-targeting drugs	[87]
Vitinoin	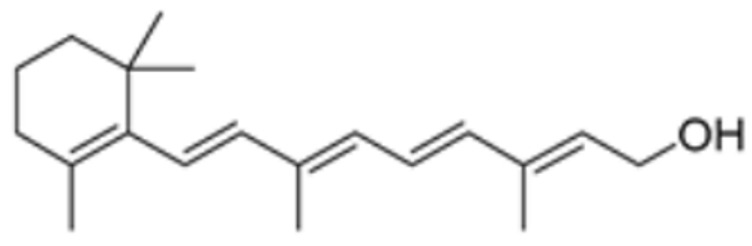	PDHA1, DBT, PDHB, DLD	/	/	219.439		[87]
Clomipramine	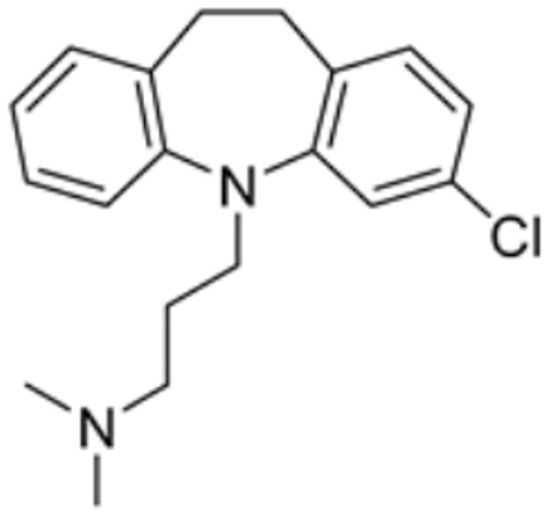	DBT, LIAS	/	/	446.560		[87]
Chlorzoxazone	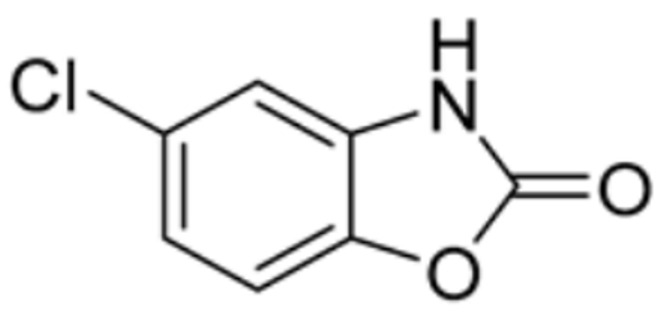	PDHA1, FDX1, DLAT, DLD	/	/	101.323		[87]
Glibenclamide	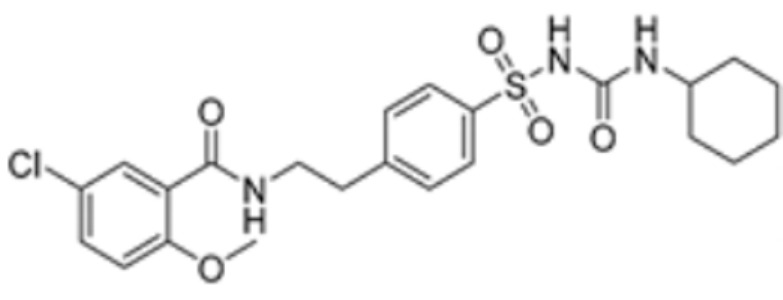	PDHA1, FDX1, DLAT, DLD	/	/	95.586		[87]
Pyruvic acid	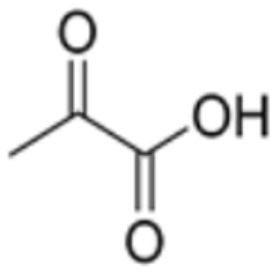	LIAS, DLD	/	/	270.716		[87]
Clindamycin	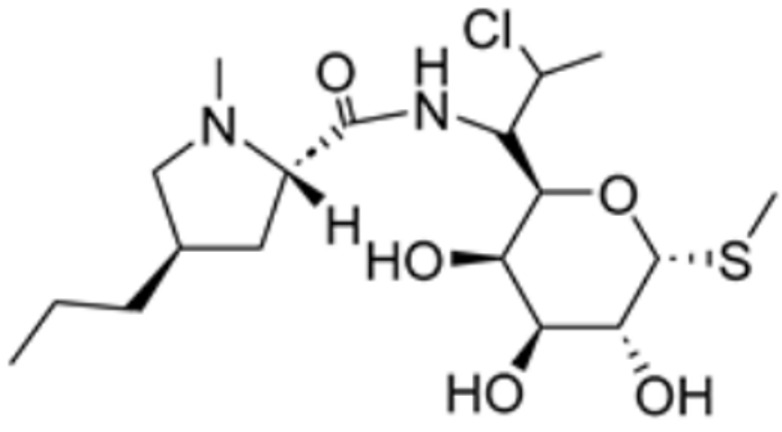	PDHA1, FDX1, DLAT, LIAS	/	/	68.639		[87]
Medrysone	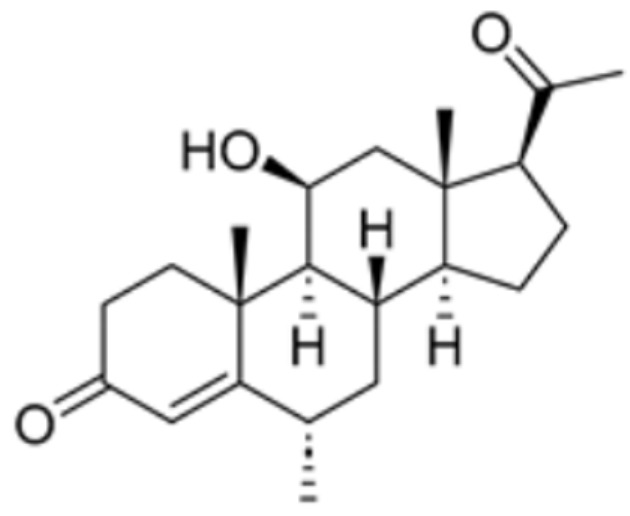	FDX1, DLAT, LIAS	/	/	90.444		[87]
Flavin adenine dinucleotide	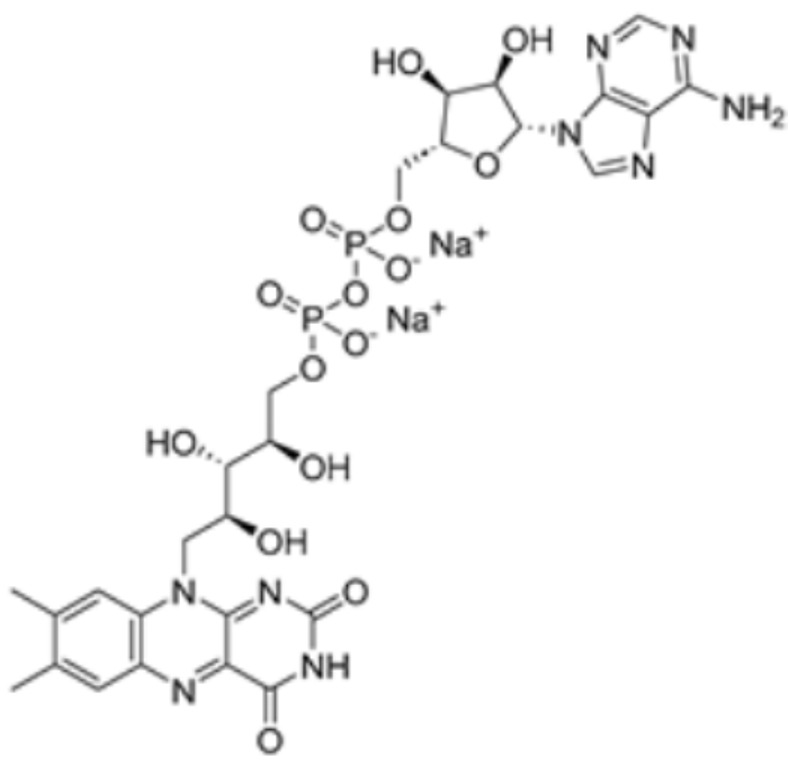	DLD	/	/	1549.437		[87]

Abbreviations: FDX1—ferredoxin 1; DLAT—dihydrolipoamide S-acetyltransferase; SLC31A1—solute carrier family 31 member 1; LIPT1—lipoyltransferase 1; DLD—dihydrolipoamide dehydrogenase; PDHB—pyruvate dehydrogenase E1 subunit β; PDHA1—pyruvate dehydrogenase E1 subunit α1; DBT—dihydrolipoamide branched chain transacylase E2; LIAS—lipoic acid synthetase.

## Data Availability

No new data were created or analyzed in this study.

## References

[1] Warnes G. (2020). Flow Cytometric Characterization of Accidental Cell Death Highlights Connections to Regulated Cell Death. J. Cell. Signal..

[2] Chen Y., Hua Y., Li X., Arslan I.M., Zhang W., Meng G. (2020). Distinct Types of Cell Death and the Implication in Diabetic Cardiomyopathy. Front. Pharmacol..

[3] Tsvetkov P., Coy S., Petrova B., Dreishpoon M., Verma A., Abdusamad M., Rossen J., Joesch-Cohen L., Humeidi R., Spangler R.D. (2022). Copper induces cell death by targeting lipoylated TCA cycle proteins. Science.

[4] Arredondo M., Núñez M.T. (2005). Iron and copper metabolism. Mol. Asp. Med..

[5] Wang Y., Zhang L., Zhou F. (2022). Cuproptosis: A new form of programmed cell death. Cell. Mol. Immunol..

[6] Chen L., Min J., Wang F. (2022). Copper homeostasis and cuproptosis in health and disease. Signal Transduct. Target. Ther..

[7] Chen L., Li N., Zhang M., Sun M., Bian J., Yang B., Li Z., Wang J., Li F., Shi X. (2021). APEX2-based Proximity Labeling of Atox1 Identifies CRIP2 as a Nuclear Copper-binding Protein that Regulates Autophagy Activation. Angew. Chem. (Int. Ed. Engl.).

[8] Braiterman L., Nyasae L., Leves F., Hubbard A.L. (2011). Critical roles for the COOH terminus of the Cu-ATPase ATP7B in protein stability, trans-Golgi network retention, copper sensing, and retrograde trafficking. Am. J. Physiol. Gastrointest. Liver Physiol..

[9] Yang H., Zhong C., Tan X., Chen G., He Y., Liu S., Luo Z. (2022). Transcriptional Responses of Copper-Transport-Related Genes ctr1, ctr2 and atox1 and Their Roles in the Regulation of Cu Homeostasis in Yellow Catfish Pelteobagrus fulvidraco. Int. J. Mol. Sci..

[10] Clifford R.J., Maryon E.B., Kaplan J.H. (2016). Dynamic internalization and recycling of a metal ion transporter: Cu homeostasis and CTR1, the human Cu^+^ uptake system. J. Cell Sci..

[11] Zhang S., Liu H., Amarsingh G.V., Cheung C.C.H., Hogl S., Narayanan U., Zhang L., McHarg S., Xu J., Gong D. (2014). Diabetic cardiomyopathy is associated with defective myocellular copper regulation and both defects are rectified by divalent copper chelation. Cardiovasc. Diabetol..

[12] Sudhahar V., Okur M.N., Bagi Z., O’bryan J.P., Hay N., Makino A., Patel V.S., Phillips S.A., Stepp D., Ushio-Fukai M. (2018). Akt2 (Protein Kinase B Beta) Stabilizes ATP7A, a Copper Transporter for Extracellular Superoxide Dismutase, in Vascular Smooth Muscle: Novel Mechanism to Limit Endothelial Dysfunction in Type 2 Diabetes Mellitus. Arterioscler. Thromb. Vasc. Biol..

[13] Jomova K., Makova M., Alomar S.Y., Alwasel S.H., Nepovimova E., Kuca K., Rhodes C.J., Valko M. (2022). Essential metals in health and disease. Chem.-Biol. Interact..

[14] Wang X., Flores S.R., Ha J.-H., Doguer C., Woloshun R.R., Xiang P., Grosche A., Vidyasagar S., Collins J.F. (2018). Intestinal DMT1 Is Essential for Optimal Assimilation of Dietary Copper in Male and Female Mice with Iron-Deficiency Anemia. J. Nutr..

[15] Rupp J.C., Locatelli M., Grieser A., Ramos A., Campbell P.J., Yi H., Steel J., Burkhead J.L., Bortz E. (2017). Host Cell Copper Transporters CTR1 and ATP7A are important for Influenza A virus replication. Virol. J..

[16] Maryon E.B., Molloy S.A., Kaplan J.H. (2013). Cellular glutathione plays a key role in copper uptake mediated by human copper transporter 1. Am. J. Physiol.-Cell Physiol..

[17] Furukawa Y., O’Halloran T.V. (2006). Posttranslational Modifications in Cu,Zn-Superoxide Dismutase and Mutations Associated with Amyotrophic Lateral Sclerosis. Antioxid. Redox Signal..

[18] Horng Y.-C., Cobine P.A., Maxfield A.B., Carr H.S., Winge D.R. (2004). Specific copper transfer from the Cox17 metallochaperone to both Sco1 and Cox11 in the assembly of yeast cytochrome C oxidase. J. Biol. Chem..

[19] Wang M., Zheng L., Ma S., Lin R., Li J., Yang S. (2023). Cuproptosis: Emerging biomarkers and potential therapeutics in cancers. Front. Oncol..

[20] Li J., Jiang Y., Xu T., Zhang Y., Xue J., Gao X., Yang X., Wang X., Jia X., Cheng W. (2020). Wilson Disease With Novel Compound Heterozygote Mutations in the *ATP7B* Gene Presenting with Severe Diabetes. Diabetes Care.

[21] Tang D., Chen X., Kroemer G. (2022). Cuproptosis: A copper-triggered modality of mitochondrial cell death. Cell Res..

[22] Lillig C.H., Berndt C., Holmgren A. (2008). Glutaredoxin systems. Biochim. Et Biophys. Acta (BBA)—Gen. Subj..

[23] Gallogly M.M., Starke D.W., Mieyal J.J. (2009). Mechanistic and Kinetic Details of Catalysis of Thiol-Disulfide Exchange by Glutaredoxins and Potential Mechanisms of Regulation. Antioxid. Redox Signal..

[24] Singleton W.C., McInnes K.T., Cater M.A., Winnall W.R., McKirdy R., Yu Y., Taylor P.E., Ke B.-X., Richardson D.R., Mercer J.F. (2010). Role of Glutaredoxin1 and Glutathione in Regulating the Activity of the Copper-transporting P-type ATPases, ATP7A and ATP7B. J. Biol. Chem..

[25] Xu W., Qian J., Hou G., Wang T., Wang J., Wang Y., Yang L., Cui X., Suo A. (2022). A Hollow Amorphous Bimetal Organic Framework for Synergistic Cuproptosis/Ferroptosis/Apoptosis Anticancer Therapy via Disrupting Intracellular Redox Homeostasis and Copper/Iron Metabolisms. Adv. Funct. Mater..

[26] Graham R.E., Elliott R.J.R., Munro A.F., Carragher N.O. (2023). A cautionary note on the use of N-acetylcysteine as a reactive oxygen species antagonist to assess copper mediated cell death. Biochemistry.

[27] Perillo B., Di Donato M., Pezone A., Di Zazzo E., Giovannelli P., Galasso G., Castoria G., Migliaccio A. (2020). ROS in cancer therapy: The bright side of the moon. Exp. Mol. Med..

[28] Husain N., Mahmood R. (2019). Copper(II) generates ROS and RNS, impairs antioxidant system and damages membrane and DNA in human blood cells. Environ. Sci. Pollut. Res..

[29] Wang W., Cui Y., Wei X., Zang Y., Chen X., Cheng L., Wang X. (2024). CuCo_2_O_4_ Nanoflowers with Multiple Enzyme Activities for Treating Bacterium-Infected Wounds via Cuproptosis-like Death. ACS Nano.

[30] Wu H., Zhang Z., Cao Y., Hu Y., Li Y., Zhang L., Cao X., Wen H., Zhang Y., Lv H. (2024). A Self-Amplifying ROS-Responsive Nanoplatform for Simultaneous Cuproptosis and Cancer Immunotherapy. Adv. Sci..

[31] Guo B., Yang F., Zhang L., Zhao Q., Wang W., Yin L., Chen D., Wang M., Han S., Xiao H. (2023). Cuproptosis Induced by ROS Responsive Nanoparticles with Elesclomol and Copper Combined with αPD-L1 for Enhanced Cancer Immunotherapy. Adv. Mater..

[32] Nalepa G., Rolfe M., Harper J.W. (2006). Drug discovery in the ubiquitin–proteasome system. Nat. Rev. Drug Discov..

[33] Zhang Z., Bi C., Schmitt S.M., Fan Y., Dong L., Zuo J., Dou Q.P. (2012). 1,10-Phenanthroline promotes copper complexes into tumor cells and induces apoptosis by inhibiting the proteasome activity. JBIC J. Biol. Inorg. Chem..

[34] Opazo C.M., Lotan A., Xiao Z., Zhang B., Greenough M.A., Lim C.M., Trytell H., Ramírez A., Ukuwela A.A., Mawal C.H. (2021). Nutrient copper signaling promotes protein turnover by allosteric activation of ubiquitin E2D conjugases. bioRxiv.

[35] Chen X., Zhang X., Chen J., Yang Q., Yang L., Xu D., Zhang P., Wang X., Liu J. (2017). Hinokitiol copper complex inhibits proteasomal deubiquitination and induces paraptosis-like cell death in human cancer cells. Eur. J. Pharmacol..

[36] Zhang P., Zhou C., Ren X., Jing Q., Gao Y., Yang C., Shen Y., Zhou Y., Hu W., Jin F. (2024). Inhibiting the compensatory elevation of xCT collaborates with disulfiram/copper-induced GSH consumption for cascade ferroptosis and cuproptosis. Redox Biol..

[37] Petersmann A., Müller-Wieland D., Müller U.A., Landgraf R., Nauck M., Freckmann G., Heinemann L., Schleicher E. (2019). Definition, Classification and Diagnosis of Diabetes Mellitus. Exp. Clin. Endocrinol. Diabetes.

[38] Ong K.L., Stafford L.K., McLaughlin S.A., Boyko E.J., Vollset S.E., Smith A.E., Dalton B.E., Duprey J., Cruz J.A., Hagins H. (2023). Global, regional, and national burden of diabetes from 1990 to 2021, with projections of prevalence to 2050: A systematic analysis for the Global Burden of Disease Study 2021. Lancet.

[39] Mikłosz A., Chabowski A. (2023). Efficacy of adipose-derived mesenchymal stem cell therapy in the treatment of chronic micro- and macrovascular complications of diabetes. Diabetes Obes. Metab..

[40] Öhrvik H., Wittung-Stafshede P. (2015). Identification of New Potential Interaction Partners for Human Cytoplasmic Copper Chaperone Atox1: Roles in Gene Regulation?. Int. J. Mol. Sci..

[41] Roberts E.A., Sarkar B. (2008). Liver as a key organ in the supply, storage, and excretion of copper. Am. J. Clin. Nutr..

[42] Ahn C., Choi J., Jeung E. (2018). Organ-specific expression of the divalent ion channel proteins NCKX3, TRPV2, CTR1, ATP7A, IREG1 and HEPH in various canine organs. Mol. Med. Rep..

[43] Kuo Y.-M., Zhou B., Cosco D., Gitschier J. (2001). The copper transporter CTR1 provides an essential function in mammalian embryonic development. Proc. Natl. Acad. Sci. USA.

[44] Flores A.G., Unger V.M. (2013). Atox1 Contains Positive Residues that Mediate Membrane Association and Aid Subsequent Copper Loading. J. Membr. Biol..

[45] Hatori Y., Lutsenko S. (2013). An Expanding Range of Functions for the Copper Chaperone/Antioxidant Protein Atox1. Antioxid. Redox Signal..

[46] Hatori Y., Lutsenko S. (2016). The role of copper chaperone Atox1 in coupling redox homeostasis to intracellular copper distribution. Antioxidants.

[47] Zou M., Zhang W., Zhu Y., Xu Y. (2023). Identification of 6 cuproptosis-related genes for active ulcerative colitis with both diagnostic and therapeutic values. Medicine.

[48] Liu M., Yu W., Jin J., Ma M., An T., Nie Y., Teng C.-B. (2020). Copper promotes sheep pancreatic duct organoid growth by activation of an antioxidant protein 1-dependent MEK-ERK pathway. Am. J. Physiol.-Cell Physiol..

[49] Ahn E.H., Kim D.W., Shin M.J., Ryu E.J., Yong J.I., Chung S.Y., Cha H.J., Kim S.J., Choi Y.J., Kim D.-S. (2016). Tat-ATOX1 inhibits streptozotocin-induced cell death in pancreatic RINm5F cells and attenuates diabetes in a mouse model. Int. J. Mol. Med..

[50] Zulkifli M., Spelbring A.N., Zhang Y., Soma S., Chen S., Li L., Le T., Shanbhag V., Petris M.J., Chen T.-Y. (2023). FDX1-dependent and independent mechanisms of elesclomol-mediated intracellular copper delivery. Proc. Natl. Acad. Sci. USA.

[51] Lu H., Zhou L., Zhang B., Xie Y., Yang H., Wang Z. (2022). Cuproptosis key gene FDX1 is a prognostic biomarker and associated with immune infiltration in glioma. Front. Med..

[52] Cai C., Zhou K., Jing J., Ren Y., Weng G., Cen D., Wang X., Huang S. (2023). Confirmation of the predictive function of cuproptosis-related gene FDX1 in clear cell renal carcinoma using qRT-PCR and western blotting. Aging.

[53] Tsvetkov P., Detappe A., Cai K., Keys H.R., Brune Z., Ying W., Thiru P., Reidy M., Kugener G., Rossen J. (2019). Mitochondrial metabolism promotes adaptation to proteotoxic stress. Nat. Chem. Biol..

[54] Cai K., Tonelli M., Frederick R.O., Markley J.L. (2017). Human Mitochondrial Ferredoxin 1 (FDX1) and Ferredoxin 2 (FDX2) Both Bind Cysteine Desulfurase and Donate Electrons for Iron–Sulfur Cluster Biosynthesis. Biochemistry.

[55] dos Santos M.C.F., Anderson C.P., Neschen S., Zumbrennen-Bullough K.B., Romney S.J., Kahle-Stephan M., Rathkolb B., Gailus-Durner V., Fuchs H., Wolf E. (2020). Irp2 regulates insulin production through iron-mediated Cdkal1-catalyzed tRNA modification. Nat. Commun..

[56] Nokhoijav E., Guba A., Kumar A., Kunkli B., Kalló G., Káplár M., Somodi S., Garai I., Csutak A., Tóth N. (2022). Metabolomic Analysis of Serum and Tear Samples from Patients with Obesity and Type 2 Diabetes Mellitus. Int. J. Mol. Sci..

[57] Lu Z., Ding L., Zhang S., Jiang X., Wang Q., Luo Y., Tian X. (2023). Bioinformatics analysis of copper death gene in diabetic immune infiltration. Medicine.

[58] Sudhahar V., Urao N., Oshikawa J., McKinney R.D., Llanos R.M., Mercer J.F., Ushio-Fukai M., Fukai T. (2013). Copper transporter ATP7A protects against endothelial dysfunction in type 1 diabetic mice by regulating extracellular superoxide dismutase. Diabetes.

[59] Abdelsaid K., Sudhahar V., Harris R.A., Das A., Liu Y., McMenamin M., Hou Y., Fulton D., Hamrick M.W., Tang Y. (2022). Exercise improves angiogenic function of circulating exosomes in type 2 diabetes: Role of exosomal SOD3. FASEB J. Off. Publ. Fed. Am. Soc. Exp. Biol..

[60] Arciello M., Longo A., Viscomi C., Capo C., Angeloni A., Rossi L., Balsano C. (2015). Core domain mutant Y220C of p53 protein has a key role in copper homeostasis in case of free fatty acids overload. BioMetals.

[61] Schweigel-Röntgen M. (2014). The Families of Zinc (SLC30 and SLC39) and Copper (SLC31) Transporters. Curr. Top. Membr..

[62] Huo S., Wang Q., Shi W., Peng L., Jiang Y., Zhu M., Guo J., Peng D., Wang M., Men L. (2023). ATF3/SPI1/SLC31A1 Signaling Promotes Cuproptosis Induced by Advanced Glycosylation End Products in Diabetic Myocardial Injury. Int. J. Mol. Sci..

[63] Zhong W., Dong Y.-J., Hong C., Li Y.-H., Xiao C.-X., Liu X.-H., Chang J. (2023). ASH2L upregulation contributes to diabetic endothelial dysfunction in mice through STEAP4-mediated copper uptake. Acta Pharmacol. Sin..

[64] Zheng X., Zhang C., Zheng D., Guo Q., Maierhaba M., Xue L., Zeng X., Wu Y., Gao W. (2023). An original cuproptosis-related genes signature effectively influences the prognosis and immune status of head and neck squamous cell carcinoma. Front. Genet..

[65] Tsymbal S.A., Refeld A.G., Kuchur O.A. (2022). The p53 Tumor Suppressor and Copper Metabolism: An Unrevealed but Important Link. Mol. Biol..

[66] Tsymbal S., Refeld A., Zatsepin V., Kuchur O. (2023). The p53 Protein is a Suppressor of Atox1 Copper Chaperon in Tumor Cells Under Genotoxic Effects. Cancer Biol..

[67] Kung C.-P., Murphy M.E. (2016). The role of the p53 tumor suppressor in metabolism and diabetes. J. Endocrinol..

[68] Si R., Zhang Q., Tsuji-Hosokawa A., Watanabe M., Willson C., Lai N., Wang J., Dai A., Scott B.T., Dillmann W.H. (2020). Overexpression of p53 due to excess protein O-GlcNAcylation is associated with coronary microvascular disease in type 2 diabetes. Cardiovasc. Res..

[69] Ma Z., Li L., Livingston M.J., Zhang D., Mi Q., Zhang M., Ding H.-F., Huo Y., Mei C., Dong Z. (2020). p53/microRNA-214/ULK1 axis impairs renal tubular autophagy in diabetic kidney disease. J. Clin. Investig..

[70] Gu J., Wang S., Guo H., Tan Y., Liang Y., Feng A., Liu Q., Damodaran C., Zhang Z., Keller B.B. (2018). Inhibition of p53 prevents diabetic cardiomyopathy by preventing early-stage apoptosis and cell senescence, reduced glycolysis, and impaired angiogenesis. Cell Death Dis..

[71] Ao H., Liu B., Li H., Lu L. (2019). Egr1 mediates retinal vascular dysfunction in diabetes mellitus via promoting p53 transcription. J. Cell. Mol. Med..

[72] Ryoo H.D., Bergmann A., Gonen H., Ciechanover A., Steller H. (2002). Regulation of Drosophila IAP1 degradation and apoptosis by reaper and ubcD1. Nat. Cell Biol..

[73] Saville M.K., Sparks A., Xirodimas D.P., Wardrop J., Stevenson L.F., Bourdon J.-C., Woods Y.L., Lane D.P. (2004). Regulation of p53 by the ubiquitin-conjugating enzymes UbcH5B/C in vivo. J. Biol. Chem..

[74] Pan C., Xiong Y., Lv X., Xia Y., Zhang S., Chen H., Fan J., Wu W., Liu F., Wu H. (2017). UbcD1 regulates Hedgehog signaling by directly modulating Ci ubiquitination and processing. EMBO Rep..

[75] Opazo C.M., Lotan A., Xiao Z., Zhang B., Greenough M.A., Lim C.M., Trytell H., Ramirez A., Ukuwela A.A., Mawal C.H. (2021). Copper Signaling Promotes Proteostasis and Animal Development Via Allosteric Activation of Ubiquitin E2 Conjugates. bioExiv.

[76] Zhang B. (2021). Regulation of Copper Homeostasis by the Ubiquitin Proteasome System. Ph.D. Thesis.

[77] Chen X., Dou Q.P., Liu J., Tang D. (2021). Targeting Ubiquitin–Proteasome System With Copper Complexes for Cancer Therapy. Front. Mol. Biosci..

[78] Kitiphongspattana K., Mathews C.E., Leiter E.H., Gaskins H.R. (2005). Proteasome Inhibition Alters Glucose-stimulated (Pro)insulin Secretion and Turnover in Pancreatic β-Cells. J. Biol. Chem..

[79] Zhou Y., Zheng Z., Wu S., Zhu J. (2023). Ubiquitin-conjugating enzyme E2 for regulating autophagy in diabetic cardiomyopathy: A mini-review. J. Diabetes.

[80] Wing S.S. (2008). The UPS in diabetes and obesity. BMC Biochem..

[81] Bugliani M., Liechti R., Cheon H., Suleiman M., Marselli L., Kirkpatrick C., Filipponi F., Boggi U., Xenarios I., Syed F. (2013). Microarray analysis of isolated human islet transcriptome in type 2 diabetes and the role of the ubiquitin–proteasome system in pancreatic beta cell dysfunction. Mol. Cell. Endocrinol..

[82] Costes S., Vandewalle B., Tourrel-Cuzin C., Broca C., Linck N., Bertrand G., Kerr-Conte J., Portha B., Pattou F., Bockaert J. (2009). Degradation of cAMP-responsive element–binding protein by the ubiquitin-proteasome pathway contributes to glucotoxicity in β-cells and human pancreatic islets. Diabetes.

[83] Chen Y., Li X., Sun R., Ji J., Yang F., Tian W., Ji W., Huang Q. (2022). A broad cuproptosis landscape in inflammatory bowel disease. Front. Immunol..

[84] Li H., Zhu N., Shi Y., Liu Q., Gu J., Qin L. (2022). Sensitivity of renal cell carcinoma to cuproptosis and cuproptosis related genes FDX1 combined with DLAT as an immunological and prognostic biomarker. Res. Sq..

[85] Guo Q., Ma M., Yu H., Han Y., Zhang D. (2023). Dexmedetomidine enables copper homeostasis in cerebral ischemia/reperfusion via ferredoxin 1. Ann. Med..

[86] Yang X., Zhang X., Shen K., Wang Z., Liu G., Huang K., He Z., Li Y., Hou Z., Lv S. (2023). Cuproptosis-related genes signature and validation of differential expression and the potential targeting drugs in temporal lobe epilepsy. Front. Pharmacol..

[87] Huang J., Zhang J., Wang F., Zhang B., Tang X. (2022). Comprehensive analysis of cuproptosis-related genes in immune infiltration and diagnosis in ulcerative colitis. Front. Immunol..

